# Supplementary material for: Dynamic changes to signal allocation rules in response to variable social environments in house mice
Source: Commun Biol. 2023 Mar 21;6:297. doi: 10.1038/s42003-023-04672-x (PMC10027867; doi:10.1038/s42003-023-04672-x)
Supplement: Supplementary file 2 — Supplementary Information [file 42003_2023_4672_MOESM2_ESM.pdf]

Supplemental Material

Supplemental Tables

Table S1. Linear mixed model details and type III analysis of variance accompanying Figure 1G (M1).

**M1 (Figure 1g) :** $\log_{10}$ (# Urine Marks + 1) ~ Recording Method + (1 | Male ID)						
**Coefficients**	**Sum Sq**	**Mean Sq**	**Num DF**	**Den DF**	**F value**	**Pr (>F)**
Recording (UV vs. Thermal)	0.00058972	0.00058972	1	430.26		0.9537

Table S2. Linear mixed model details and type III analysis of variance accompanying Figure 2 (M2-M4).

**M2 (Figure 2a) :** $\log_{10}$ (# Urine Marks + 1) ~ Fight Outcome * Mesh Trial + (1 | Male ID)						
**Coefficients**	**Sum Sq**	**Mean Sq**	**Num DF**	**Den DF**	**F value**	**Pr (>F)**
Mesh Trial (Mesh 1 vs. Mesh 2)	0.31856	0.31856	1	60.327	1.5206	0.222310
Fight Outcome (Win vs. Loss)	2.14388	2.14388	1	67.695	10.2337	0.002099 **
Mesh Trial : Fight Outcome	2.47399	2.47399	1	60.327	11.8094	0.001073 **
**M3 (Figure 2c) :** $\log_{10}$ (# Mesh 2 Marks + 1) ~ Fight Outcome + $\log_{10}$ (# Mesh1 Marks) + (1 | Strain)						
**Coefficients**	**Sum Sq**	**Mean Sq**	**Num DF**	**Den DF**	**F value**	**Pr (>F)**
Fight Outcome (Win vs. Loss)	5.1665	5.1665	1	56.73	15.0200	0.0002784 ***
Initial Investment *(Mesh1 Marks)*	3.1626	3.1626	1	58.534	9.1945	0.0036139 **
**M4 (Figure 2e) :** $\log_{10}$ (Mark Latency + 0.08) ~ Fight Outcome * Mesh Trial * $\log_{10}$ (# Mesh1 Marks) + (1 | Male ID)						
**Coefficients**	**Sum Sq**	**Mean Sq**	**Num DF**	**Den DF**	**F value**	**Pr (>F)**
Mesh Trial (Mesh 1 vs. Mesh 2)	3.3690	3.3690	1	58.141	10.3995	0.0020699 **
Fight Outcome (Win vs. Loss)	0.0835	0.0835	1	57.208	0.2579	0.6135265
Initial Investment (Mesh1 Marks)	12.0187	12.0187	1	59.120	37.0998	9.071e-08 ***
Mesh Trial : Fight Outcome	1.2604	1.2604	1	58.141	3.8907	0.0533173 .
Mesh Trial : Initial Investment	2.9995	2.9995	1	58.141	9.2589	0.0035146 **
Fight Outcome : Initial Investment	0.2482	0.2482	1	59.834	0.7661	0.3849309
Mesh Trial : Fight Outcome : Initial Investment	3.9212	3.9212	1	58.141	12.1042	0.0009601 ***

Table S3. Linear mixed model details and type III analysis of variance accompanying Figure 3 (M5-M6).

**M5 (Figure 3c) :** $\log_{10}$ (Within-Bout IMI + 0.0005) ~ Fight Outcome * Mesh Trial + (1 | BoutID) + (1 | MaleID)						
**Coefficients**	**Sum Sq**	**Mean Sq**	**Num DF**	**Den DF**	**F value**	**Pr (>F)**
Mesh Trial (Mesh 1 vs. Mesh 2)	31.777	31.777	1	428.08	304.0266	<2e-16 ***
Fight Outcome (Win vs. Loss)	0.008	0.008	1	46.72	0.0793	0.7795
Mesh Trial : Fight Outcome	0.129	0.129	1	416.35	1.2359	0.2669
**M6 (Figure 3e) :** $\log_{10}$ (Avg Marks per Bout) ~ Fight Outcome * Mesh Trial + (1 | MaleID)						
**Coefficients**	**Sum Sq**	**Mean Sq**	**Num DF**	**Den DF**	**F value**	**Pr (>F)**
Mesh Trial (Mesh 1 vs. Mesh 2)	0.004782	0.004782	1	58.447	0.2391	0.626681
Fight Outcome (Win vs. Loss)	0.204404	0.204404	1	58.831	10.2206	0.002236 **
Mesh Trial : Fight Outcome	0.025001	0.025001	1	58.447	1.2501	0.268106

Table S4. Linear mixed model details and type III analysis of variance accompanying Figure 4 (M7-M9).

**M7 (Figure 4a) :** log_{10} (# Urine Marks + 1) ~ Fight Outcome * Trial + log_{10} (# Mesh1 Marks) + (1 | MaleID)						
**Coefficients**	**Sum Sq**	**Mean Sq**	**Num DF**	**Den DF**	**F value**	**Pr (>F)**
Trial Type (Empty, S-S, S-FM, S-UM, FM-UM)	1.0377	0.25943	4	76.328	5.2319	0.0008878 ***
Fight Outcome (Win vs. Loss)	1.3221	1.3221	1	83.380	26.6632	1.62e-06 ***
Initial Investment (Mesh1 Marks)	1.6109	1.6109	1	58.040	32.4870	4.23e-07 ***
Trial : Fight Outcome	1.1442	0.28605	4	76.591	5.7686	0.0004115 ***
**M8 (Figure 4c) :** log_{10} (# Marked – Empty Trial Marks + 1) ~ Fight Outcome * Trial Group						
**Coefficients**	**Sum Sq**	**Mean Sq**	**Num DF**	**Den DF**	**F value**	**Pr (>F)**
Fight Outcome (Win vs. Loss)	0.6872	0.6872	1	58	0.6426	0.4260338
Trial Group (UM vs. no UM)	0.4194	0.4194	1	58	0.3922	0.5336060
Fight Outcome : Trial Group	14.355	14.3554	1	58	13.4233	0.0005399 ***
**M9 (Figure 4e) :** log_{10} (Mark Latency + 1) Fight Outcome + log_{10} (# Mesh1 Marks) + Trial Group + [Fight Outcome * Trial Group] + [Fight Outcome * log_{10} (# Mesh1 Marks)] + (1 | MaleID)						
**Coefficients**	**Sum Sq**	**Mean Sq**	**Num DF**	**Den DF**	**F value**	**Pr (>F)**
Fight Outcome (Win vs. Loss)	0.5932	0.5932	1	55.993	1.3338	0.253036
Trial Group (UM vs. no UM)	2.8150	1.4075	2	74.765	3.1646	0.047942 *
Initial Investment (Mesh1 Marks)	4.4721	4.4721	1	56.820	10.0549	0.002449 **
Fight Outcome : Trial Group	1.2980	0.6490	2	74.297	1.4592	0.239013
Fight Outcome : Initial Investment	1.6656	1.6656	1	57.256	3.7448	0.057917 .

Table S5. Linear mixed model details and type III analysis of variance accompanying Figure 5 (M10-M11).

**M10 (Figure 5b) :** log_{10} (Within-Bout IMI + 0.0005) ~ Fight Outcome * Trial Group + (1 | BoutID) + (1 | MaleID)						
**Coefficients**	**Sum Sq**	**Mean Sq**	**Num DF**	**Den DF**	**F value**	**Pr (>F)**
Fight Outcome (Win vs. Loss)	0.7495	0.7495	1	64.443	6.1962	0.01539 *
Trial Group (UM vs. no UM)	9.6363	4.8182	2	152.158	39.8347	1.223e-14 ***
Fight Outcome : Trial Group	0.6107	0.3053	2	153.744	2.5245	0.08342 .
**M11 (Figure 5e) :** log_{10} (Avg Marks per Bout) ~ Fight Outcome * Trial Group + (1 | MaleID)						
**Coefficients**	**Sum Sq**	**Mean Sq**	**Num DF**	**Den DF**	**F value**	**Pr (>F)**
Fight Outcome (Win vs. Loss)	0.37856	0.37856	1	66.205	22.4383	1.189e-05 ***
Trial Group (UM vs. no UM)	0.44078	0.22039	2	75.532	13.0634	1.342e-05 ***
Fight Outcome : Trial Group	0.21121	0.10561	2	75.532	6.2597	0.003052 **

Supplemental Material

Supplemental Figures

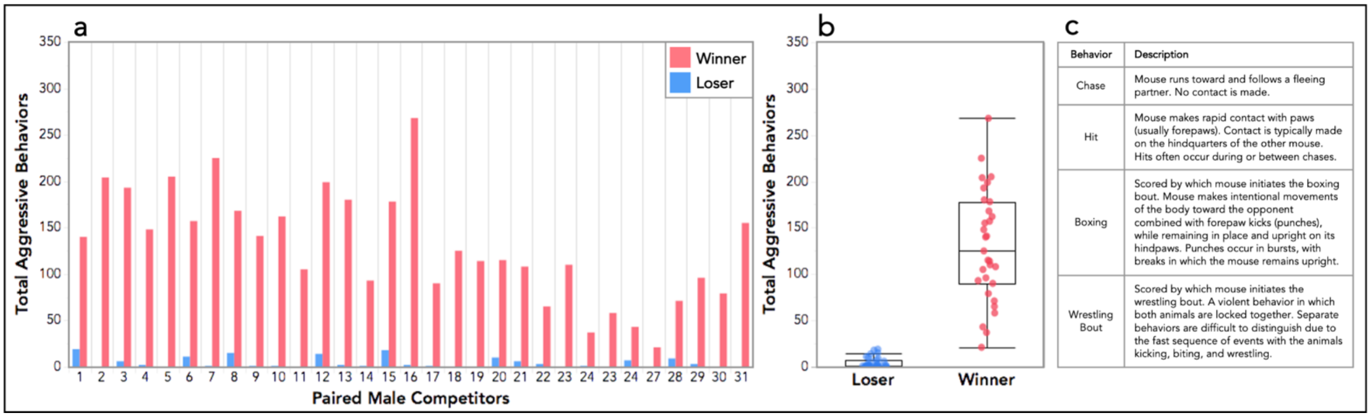

Behavior	Description
Chase	Mouse runs toward and follows a fleeing partner. No contact is made.
Hit	Mouse makes rapid contact with paws (usually forepaws). Contact is typically made on the hindquarters of the other mouse. Hits often occur during or between chases.
Boxing	Scored by which mouse initiates the boxing bout. Mouse makes intentional movements of the body toward the opponent combined with forepaw kicks (punches), while remaining in place and upright on its hindpaws. Punches occur in bursts, with breaks in which the mouse remains upright.
Wrestling Bout	Scored by which mouse initiates the wrestling bout. A violent behavior in which both animals are locked together. Separate behaviors are difficult to distinguish due to the fast sequence of events with the animals kicking, biting, and wrestling.

Figure S1. Male aggressive behaviors scored in contests (fight trials) between paired competitors. **(a)** Total aggressive behaviors performed by each paired male competitor. The fight outcome (the categorization of winners and losers) was determined by which male performed more aggressive behaviors within a pair. **(b)** Across all 31 pairs, winning males performed considerably more aggressive behaviors than losing males. Boxplot midline: median, box limits: upper and lower quartiles, whiskers: 1.5x interquartile range, points: outliers. **(c)** Behavioral ethogram used to score aggressive behaviors. State events: chase, boxing and wrestling bouts. Points events: hits. All events were coded for a male subject if the individual initiated the behavior (i.e. wrestling bout is coded for only one participant – the initiator – of that event).

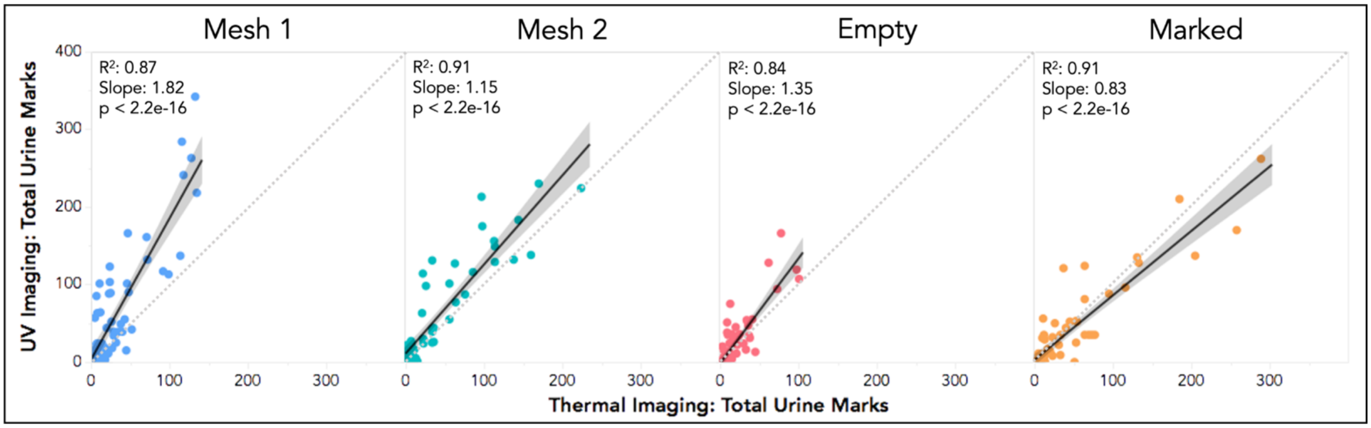

Figure S2. Comparison of urine mark detection methods across trial types: Ultraviolet light (UV) blot imaging vs. thermal imaging. The two detection methods are well-correlated with each other (R > 0.8). For both Mesh trials and the Empty trials, UV imaging consistently detected more urine marks than thermal imaging. The Marked trials revealed the opposite pattern, with thermal imagining detecting more urine marks than UV imaging. This is likely due to the challenge of detection marks placed on top of the aliquoted urine placed in the arena at the beginning of each Marked trial. Three trials were excluded from this dataset due to poor urine blot quality, and one trial was excluded as an outlier.

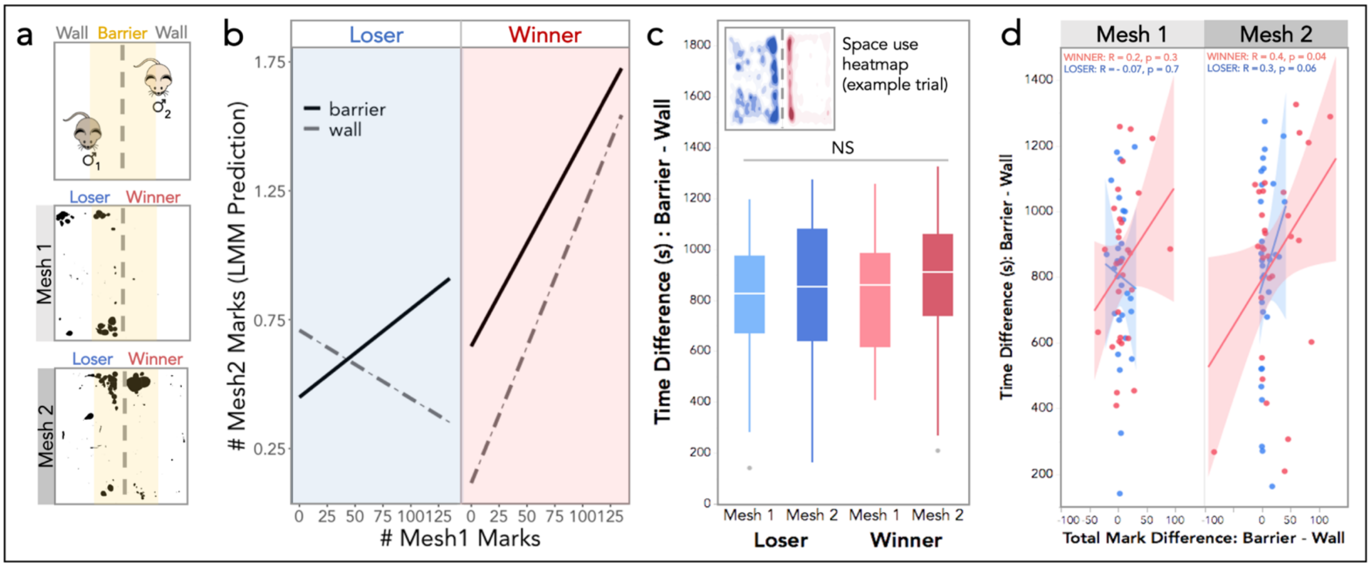

Figure S3. Mesh trial spatial marking and space use. (a) Top: schematic of the mesh trials indicating the social "Barrier" (yellow) and non-social "Wall" regions of interest (ROIs). Below: Example mesh trial urine blots of a male pair (winner and loser) pre- and post-fight demonstrating the spatial allocation of urine marks at the social boundary. **(b)** Estimated marginal means plot for the total number of marks in the post-fight mesh trial (Mesh 2) given the fight outcome (winner: red, loser: blue), initial signal investment (# Mesh 1 marks), and the ROI (Barrier: solid, Wall: dashed). **(c)** Difference in time (s) spent in the Barrier vs. Wall regions of interest (ROIs) across mesh trials by winning and losing males. Winners and losers spend more time at the social boundary (Barrier) across mesh trials. Top left corner: an example heat map of a male pair in a mesh trial (Mesh 1), depicting how all males spend more time at the social boundary (Barrier) than the non-social ROI (Wall) across mesh trials, regardless of fight outcome. Boxplot midline: median, box limits: upper and lower quartiles, whiskers: 1.5x interquartile range, points: outliers. **(d)** Comparison of the difference in time spent vs. the difference in total marks allocated in the ROIs (Barrier – Wall) by winners and loser across trials. In both mesh trials, space use and changes in urine allocation effort are not detectably correlated with each other among winning or losing males (R < 0.2). **(b-d)** Linear mixed models were used to model relationships, analyses of variance were used to test for overall effects and post hoc pairwise comparisons were performed using the *emmeans* package (significance code: NS p>0.05).

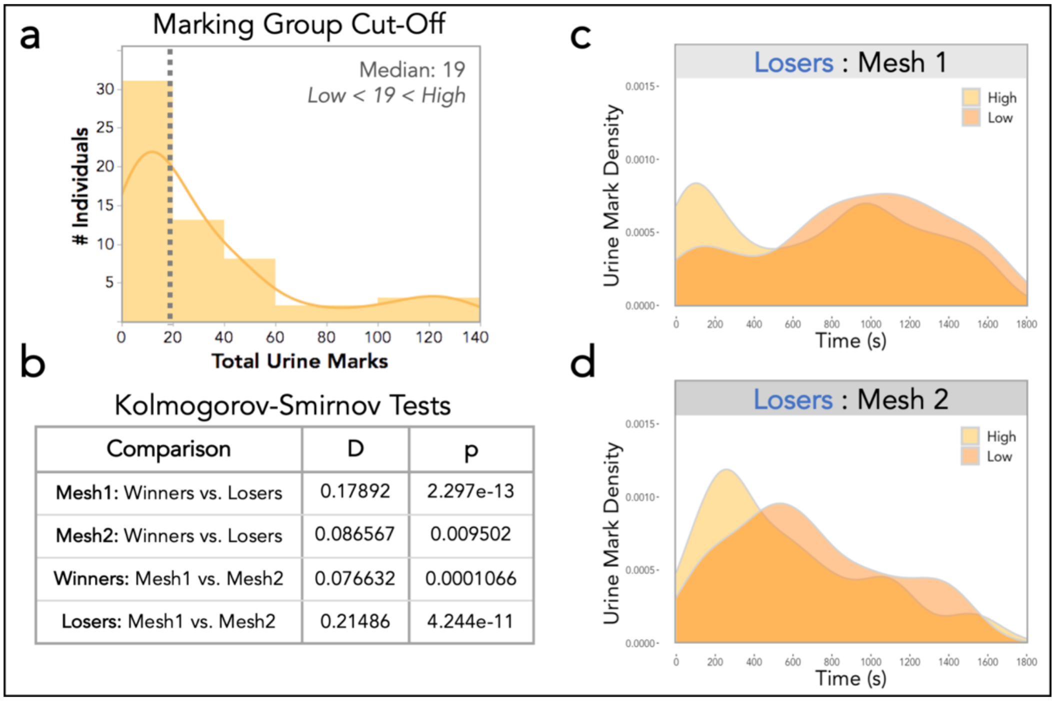

Comparison	D	p
**Mesh1:** Winners vs. Losers	0.17892	2.297e-13
**Mesh2:** Winners vs. Losers	0.086567	0.009502
**Winners:** Mesh1 vs. Mesh2	0.076632	0.0001066
**Losers:** Mesh1 vs. Mesh2	0.21486	4.244e-11

Figure S4. Comparisons of temporal distributions of urine mark deposition. (a) Males were separated into two groups (high and low-marking) based on whether the number of marks deposited pre-fight (Mesh 1) fell above or below the median number of marks (19: dashed line). Histogram of the distribution of total urine marks deposited by all males competitors in the Mesh 1 (pre-fight). This categorization was used to examine differences in mark distribution across low and high-marking losers (C-D). **(b)** Kolmogorov-Smirnov two-sample statistical tests comparing the distributions of scent marking across the trial duration depicted in Figure 2d. **(c-d)** Density plots depicting urine deposition events over the two 30-minute mesh trials (Mesh 1: top, Mesh 2: bottom) for losing males that were either initially high-marking (yellow) or low-marking (orange). During the first mesh trial high marking losers are contributing most to the early peak ~150s, while low-marking losers don't peak in marking activity until ~1000s. This dramatic difference diminishes by the second mesh trial.

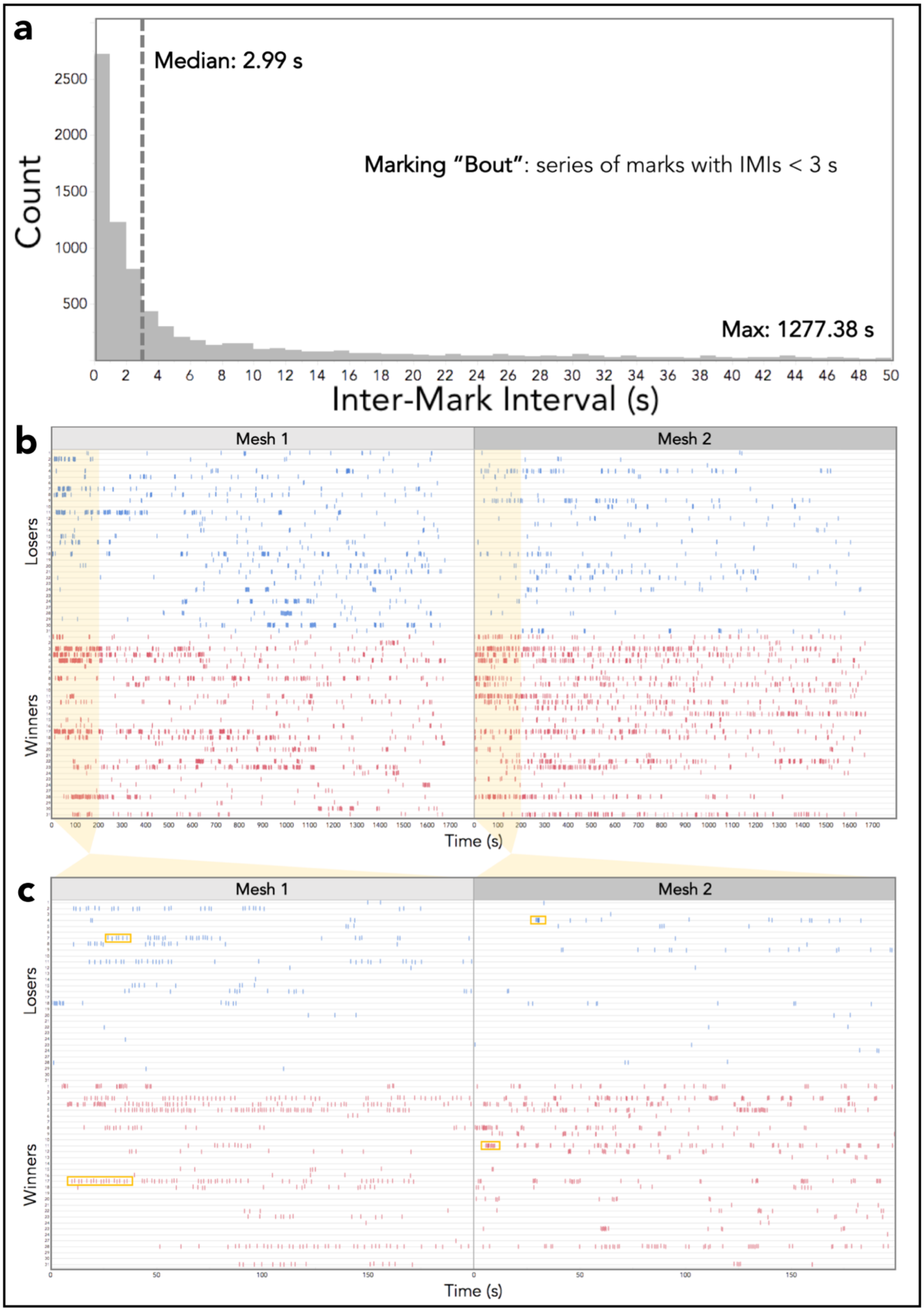

Figure S5. Timing of urine deposition events in mesh trials. (a) Histogram of the inter-mark intervals (IMIs) for all males across all trials. The median value is indicated with a dashed line (2.99 seconds). The range of IMIs extends to nearly the full trial length (only the first 50s is shown), The maximum values are reported in the bottom right corner. The median IMI value was used to define a marking "bout." Such that any two marks that occur in sequence with an IMI < 3 seconds are grouped together into a multi-mark bout. **(b)** Event plots depicting the urine marking of all male competitors over the course of both mesh trials (Mesh 1=left, Mesh 2 = right) for the entire trial duration (1800 seconds). Pair IDs are indicated on the left-hand axis. Losers depicted on top in blue, and winners on the bottom in red. **(c)** Event plots depicted for a zoomed-in view of the first 200 seconds of the trials for all individuals. Example "chain"-like bouts are outlined in the Mesh 1 panel, and example "burst"-like marking bouts are highlighted in the Mesh 2 panel (yellow boxes).

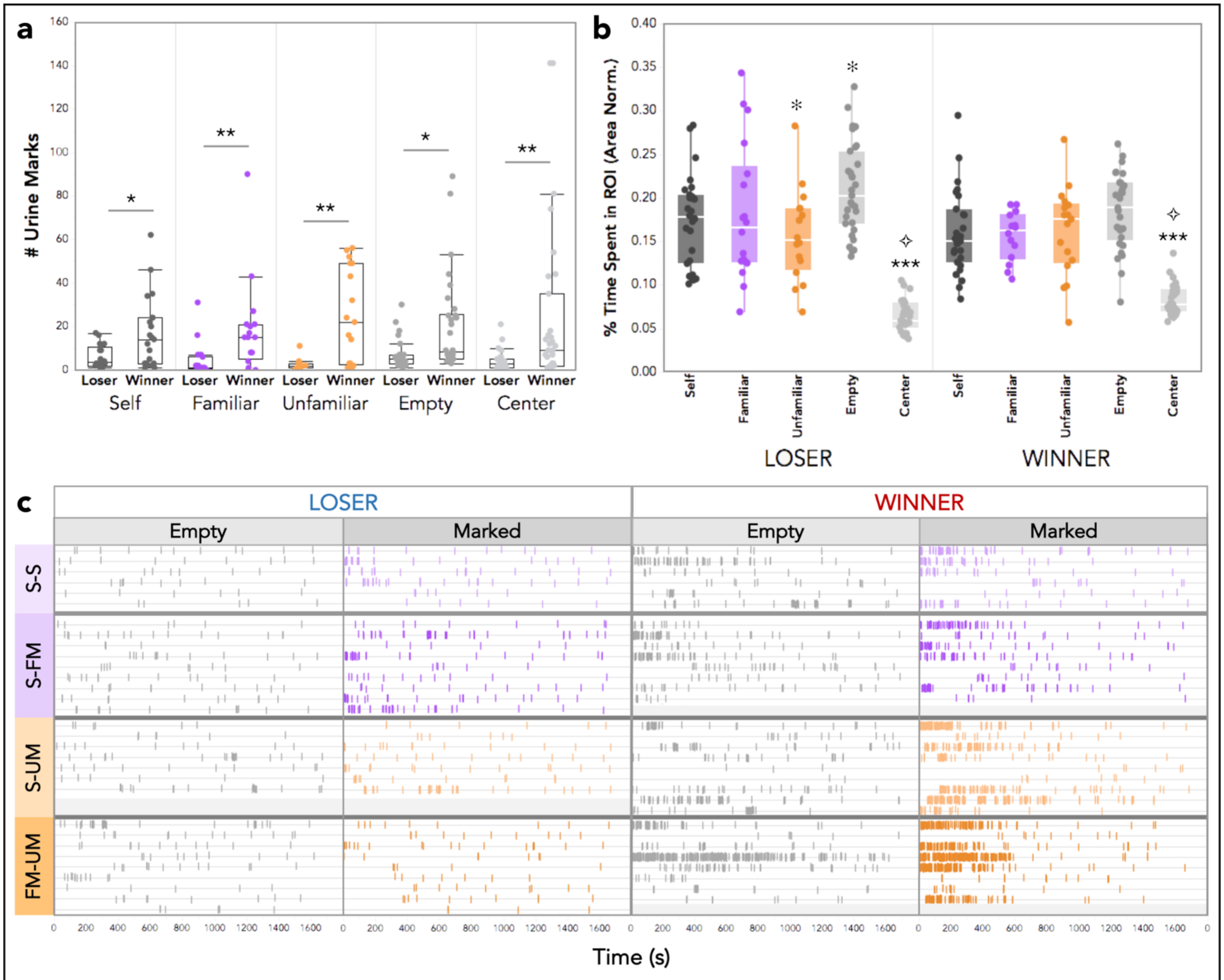

d

Familiarity & Fight Outcome			*Scent-Marked Environment & Fight Outcome*		
Two-Sided Comparisons	D	p	Two-Sided Comparisons	D	p
No Unfamiliar : Winners vs. Losers	0.17906	1.169e-06	Winners : S-S vs. S-FM	0.12509	0.04124
Unfamiliar : Winners vs. Losers	0.47886	< 2.2e-16	Winners : S-UM vs. FM-UM	0.18929	5.773e-15
Winners : No Unfamiliar vs. Unfamiliar	0.12235	1.302e-05	Losers : S-S vs. S-FM	0.028598	0.9991
Losers : No Unfamiliar . Unfamiliar	0.25871	3.244e-08	Losers : S-UM vs. FM-UM	0.23213	0.009006

Figure S6. Urine deposition in scent-marked trials. (a) Total number of marks deposited by winners and losers in scent-marked trials to specific ROIs: scent-marked corners (containing self, familiar, or unfamiliar male urine), empty corners, or the center of the arena (significance codes: * $p<0.05$; ** $p<0.01$). **(b)** The percent of time spent in specific urine-marked trial ROIs by winners and losers, normalized to the total area of each ROI (to account for the center being larger). Winners and losers spend significantly less time in the center ROI than all corner ROIs (Self, Familiar, Unfamiliar or Empty; *** $p<0.001$). Losers spend significantly less time in the center ROI than winners (✧ $p<0.01$). Losers spend significantly less time in corners with unfamiliar urine relative to empty ones (✻ $p<0.05$). **(a,b)** Boxplot midline: median, box limits: upper and lower quartiles, whiskers: 1.5x interquartile range, points: outliers. Linear mixed models were used to model relationships, analyses of variance were used to test for overall effects, and post hoc pairwise comparisons were performed using the *emmeans* package (see Data Availability for code) **(c)** Event plots depicting the urine marking of winning and losing males to the empty trials and the urine-marked trials for the entire trial duration (1800 seconds). Males are grouped by the four different scent-marked treatments: self-self (S-S: light purple), self-familiar male (S-FM: dark purple), self-unfamiliar male (S-UM: light orange), and familiar male-unfamiliar male (FM-UM: dark orange). Almost all male pairs experienced the same treatment, three pairs received different urine-marked treatments due to urine stimuli collection constraints (hence some of the treatment groups have unequal paired number across fight outcome groupings). **(d)** Kolmogorov-Smirnov two-sample statistical tests comparing the distributions of scent marking across the trial durations depicted in Figure 4d. Comparisons were by trial groups (No Unfamiliar vs. Unfamiliar), as well as for specific scent-marked treatments (S-S, S-FM, S-UM & FM-UM).
