# Supplementary material for: Dynamic changes to signal allocation rules in response to variable social environments in house mice
Source: Commun Biol. 2023 Mar 21;6:297. doi: 10.1038/s42003-023-04672-x (PMC10027867; doi:10.1038/s42003-023-04672-x)
Supplement: Supplementary file 3 — Description of Additional Supplementary Files [file 42003_2023_4672_MOESM3_ESM.pdf]

Description of Additional Supplementary Files

File name: Supplemental Data
Description: Datasheets used in analyses, summary datasheets & annotated R code.

File name: Video S1
Description: Thermal video recording of an example mesh trial (Days 1 & 3 of the trial series; Figure 1A), in which two male competitors are separated by a mesh barrier. Urine scent marking can be observed as thermal hot spots (pink) deposited by males as they traverse the environment. These hot spots will subsequently cool below substrate temperatures (dark blue) as the urine cools.

File name: Video S2
Description: Thermal video recording of an example urine scent-marked trial (Day 4 of the trial series; Figure 1A). Scent-marked zones of aliquoted urine stimuli are visible as cold spots (dark blue) in two corners of the arena. Urine marking of the focal male can be observed as thermal hot spots (pink) deposited as the male traverses the arena. These hot spots will subsequently cool below substrate temperatures (dark blue) as the urine cools.
