# Supplementary material for: Dynamic changes to signal allocation rules in response to variable social environments in house mice
Source: Commun Biol. 2023 Mar 21;6:297. doi: 10.1038/s42003-023-04672-x (PMC10027867; doi:10.1038/s42003-023-04672-x)
Supplement: Supplementary file 7 — Reporting Summary [file 42003_2023_4672_MOESM7_ESM.pdf]

nature portfolio

Corresponding author(s): Caitlin H Miller; Michael J Sheehan

Last updated by author(s): Dec 8, 2022

Reporting Summary

Nature Portfolio wishes to improve the reproducibility of the work that we publish. This form provides structure for consistency and transparency in reporting. For further information on Nature Portfolio policies, see our Editorial Policies and the Editorial Policy Checklist.

Statistics

For all statistical analyses, confirm that the following items are present in the figure legend, table legend, main text, or Methods section.

n/a	Confirmed	
☐	☒	The exact sample size (*n*) for each experimental group/condition, given as a discrete number and unit of measurement
☐	☒	A statement on whether measurements were taken from distinct samples or whether the same sample was measured repeatedly
☐	☒	The statistical test(s) used AND whether they are one- or two-sided<br>*Only common tests should be described solely by name; describe more complex techniques in the Methods section.*
☐	☒	A description of all covariates tested
☐	☒	A description of any assumptions or corrections, such as tests of normality and adjustment for multiple comparisons
☐	☒	A full description of the statistical parameters including central tendency (e.g. means) or other basic estimates (e.g. regression coefficient) AND variation (e.g. standard deviation) or associated estimates of uncertainty (e.g. confidence intervals)
☐	☒	For null hypothesis testing, the test statistic (e.g. *F*, *t*, *r*) with confidence intervals, effect sizes, degrees of freedom and *P* value noted<br>*Give P values as exact values whenever suitable.*
☒	☐	For Bayesian analysis, information on the choice of priors and Markov chain Monte Carlo settings
☐	☒	For hierarchical and complex designs, identification of the appropriate level for tests and full reporting of outcomes
☒	☐	Estimates of effect sizes (e.g. Cohen's *d*, Pearson's *r*), indicating how they were calculated

Our web collection on statistics for biologists contains articles on many of the points above.

Software and code

Policy information about availability of computer code

Data collection	NA
Data analysis	R code used in analyses are included (Supplemental Data).

For manuscripts utilizing custom algorithms or software that are central to the research but not yet described in published literature, software must be made available to editors and reviewers. We strongly encourage code deposition in a community repository (e.g. GitHub). See the Nature Portfolio guidelines for submitting code & software for further information.

Data

Policy information about availability of data

All manuscripts must include a data availability statement. This statement should provide the following information, where applicable:

- Accession codes, unique identifiers, or web links for publicly available datasets
- A description of any restrictions on data availability
- For clinical datasets or third party data, please ensure that the statement adheres to our policy

All data used in data analysis as well as summary files are included (as CSV files).

Human research participants

Policy information about studies involving human research participants and Sex and Gender in Research.

Reporting on sex and gender	NA
Population characteristics	NA
Recruitment	NA
Ethics oversight	NA

Note that full information on the approval of the study protocol must also be provided in the manuscript.

Field-specific reporting

Please select the one below that is the best fit for your research. If you are not sure, read the appropriate sections before making your selection.

☒ Life sciences ☐ Behavioural & social sciences ☐ Ecological, evolutionary & environmental sciences

For a reference copy of the document with all sections, see nature.com/documents/nr-reporting-summary-flat.pdf

Life sciences study design

All studies must disclose on these points even when the disclosure is negative.

Sample size	A total of 62 males were used. Males were placed into 2 dominance groups (winner vs. loser: 31 males per group). Males were subsequently placed into one of four possible urine stimulus treatment groups on the 4th trial day (self-self, self-familiar male, self-unfamiliar male, familiar male-unfamiliar male). Each dominance x treatment subgroup (e.g. losing male subjected to self-self treatment) consisted of 6-9 males. Originally we had planned to test marking responses differences to the 2 stimuli within treatments. However, upon further analysis and with a greater sample size it became clear that the space in the arena was insufficient to observe spatially distinct marking patterns to the 2 different urine stimuli zones. Nevertheless, we observe statistically robust responses to the treatment groups, as an entire scent environment. We further pooled the pairs of similar treatment groups in subsequent analyses to increase our sample size for more robust statistical testing.
Data exclusions	The only data exclusions were performed in the recording methods comparisons Figure S2. Three trials were excluded from this dataset due to poor urine blot quality, and one trial was excluded as an outlier. These males were not excluded from any other analyses anywhere else in the manuscript.
Replication	This experimental design was intentionally built off of a previous and well-established experimental design (Desjardins, Maruniak & Bronson, 1973). Our study expanded on this design by adding 2 additional trial days, added novel recording methodology, and a greater sample size. We find overall similar patterns. However, the addition of temporal marking data, and pairing well-matched males, provided new insights.
Randomization	Male trial order and arena chamber was pseudo-randomized each day to avoid confounds in arena location and marking behavior over the course of the designated trial period. The orientation within the Mesh 1 trials was also randomized (whether males were placed near the back or front of the arena) to account for variation in sound disturbances for males closer to the chamber door; orientations were subsequently flipped for each pair in Mesh 2. Urine-marked trial treatments were pseudo-randomly assigned to each male pair, to ensure similar numbers of male pairs were exposed to the 4 treatment types across sets of trials series. The orientation of urine stimuli was randomly assigned to corner orientations (front right – back left vs. back right – front left). Lastly, the fur bleaching for male identification was performed on one mouse strain (NY2 or NY3) for each trial set, but the bleached strain was switched between trial sets to prevent errors within a trial set and to avoid bleaching only one strain across trial sets.
Blinding	All scoring was performed by a blind observer.

Reporting for specific materials, systems and methods

We require information from authors about some types of materials, experimental systems and methods used in many studies. Here, indicate whether each material, system or method listed is relevant to your study. If you are not sure if a list item applies to your research, read the appropriate section before selecting a response.

Materials & experimental systems

n/a	Involved in the study	
☒	☐	Antibodies
☒	☐	Eukaryotic cell lines
☒	☐	Palaeontology and archaeology
☐	☒	Animals and other organisms
☒	☐	Clinical data
☒	☐	Dual use research of concern

Methods

n/a	Involved in the study	
☒	☐	ChIP-seq
☒	☐	Flow cytometry
☒	☐	MRI-based neuroimaging

Animals and other research organisms

Policy information about studies involving animals; ARRIVE guidelines recommended for reporting animal research, and Sex and Gender in Research

Laboratory animals	Laboratory house mice (Mus musculus domesticus) were used in this study. Two mouse strains were used as experimental individuals: two wild-derived inbred strains (NY2 and NY3). The progenitors of these strains were captured near Saratoga Springs, NY in 2013 by MJS and are related to the SarA/NachJ, SarB/NachJ and SarC/NachJ strains now available from the Jackson Lab. The urine of a third mouse strain (C57BL/6J) was used as an unfamiliar male stimulus.
Wild animals	NA
Reporting on sex	All mice used in this study were male, as we were interested in urine scent mark signaling in responses to competition. This signaling modality is performed most clearly in males within a competitive context.
Field-collected samples	NA
Ethics oversight	All experimental protocols conducted at Cornell University were approved by the Institutional Animal Care and Use Committee (IACUC: Protocol #2015-0060) and were in compliance with the NIH Guide for Care and Use of Animals.

Note that full information on the approval of the study protocol must also be provided in the manuscript.
